# Persistent homology reveals robustness loss in inhaled substance abuse rs-fMRI networks

**DOI:** 10.1371/journal.pone.0310165

**Published:** 2024-09-16

**Authors:** Martin Mijangos, Lucero Pacheco, Alessandro Bravetti, Nadia González-García, Pablo Padilla, Roberto Velasco-Segura

**Affiliations:** 1 Instituto de Investigaciones en Matemáticas Aplicadas y en Sistemas, Universidad Nacional Autónoma de México, Mexico City, Mexico; 2 Facultad de Medicina, Universidad Nacional Autónoma de México, Mexico City, Mexico; 3 Laboratorio de Neurociencias, Hospital Infantil de México Federico Gómez, Mexico City, Mexico; 4 Instituto de Ciencias Aplicadas y Tecnología, Universidad Nacional Autónoma de México, Mexico City, Mexico; Museo Storico della Fisica e Centro Studi e Ricerche Enrico Fermi, ITALY

## Abstract

Analyzing functional brain activity through functional magnetic resonance imaging (fMRI) is commonly done using tools from graph theory for the analysis of the correlation matrices. A drawback of these methods is that the networks must be restricted to values of the weights of the edges within certain thresholds and there is no consensus about the best choice of such thresholds. Topological data analysis (TDA) is a recently-developed tool in algebraic topology which allows us to analyze networks through combinatorial spaces obtained from them, with the advantage that all the possible thresholds can be considered at once. In this paper we applied TDA, in particular persistent homology, to study correlation matrices from rs-fMRI, and through statistical analysis, we detected significant differences between the topological structures of adolescents with inhaled substance abuse disorder (ISAD) and healthy controls. We interpreted the topological differences as indicative of a loss of robustness in the functional brain networks of the ISAD population.

## Introduction

Neuroimaging is a commonly-used tool to study different aspects of brain structure [1] and function [2]. Resting-state magnetic functional resonance imaging (rs-fMRI) has been used to study the co-activation of brain regions. The time series of the blood oxygen level dependent (BOLD) signal is used to calculate a correlation matrix between selected regions, which serves as an adjacency matrix. These networks are then usually analyzed with techniques from graph theory [3]. In order to study global topological properties of the networks that capture high-dimensional relations between the nodes, simplicial complexes have been constructed from these graphs [4]. An issue with weighted networks is that they must be binarized for applying these constructions, and there is no consensus about how to choose an optimal threshold for this step [5]. Here is where Topological Data Analysis (TDA) is extremely useful: on the one hand it allows to analyze the network considering different scales at once, and on the other hand it focuses on the global properties of the network. In particular, persistent homology, the most common tool in TDA, has been applied to the study of functional brain networks. This approach was first put forward in [6] and has gained a lot of attention ever since. For instance, Petri, et al. [7] analyzed networks coming from fMRI of healthy controls and people under the influence of psilocybin; in [8] the activity of pyramidal neurons in rat hippocampus was analyzed; in [9] the focus has been on brain networks obtained from diffusion spectrum imaging, with the aim of identifying topological cavities which represent relevant architectural features of the human connectome; in [10] the authors showed that these techniques can be used to provide empirical evidence for decreased functional integration in patients affected by Alzheimer’s disease; in [11] the authors reported the discovery of topological phase transitions in functional brain networks. In [12] these networks are studied in order to identify differences between the brain topology of typically-developing children and that of children with attention-deficit/hyperactivity disorder; finally, we refer to [13] for a recent accessible introduction to TDA and its applications to the analysis of multivariate time series data, including interesting open problems in neuroscience. An open problem that has never been addressed so far in the literature is comparing the topological structure of functional brain networks of subjects with inhaled substance abuse disorder (ISAD) and healthy controls. This is the aim of the present work.

Another well-known issue with the application of TDA to real data is that the results of persistent homology are commonly given in the form of barcodes, a sort of fingerprint of the network; however, extracting useful information from the barcodes is not an easy task. To resolve this problem, a number of techniques have been developed, such as persistence landscapes [14], Betti curves [15], persistent entropy [16], or simply the analysis of the statistics of the lengths of the bars [17].

In this paper we focus on two aspects: first, we propose a pipeline to apply persistent homology to networks obtained from rs-fMRI, and then we show by a statistical analysis that the persistent entropy is a good indicator of the differences in the thus-obtained topological information. The first (topological) part of the work is based on the construction in [8]. However, we allow the networks to have edges with the same weight. In the second (statistical) part of the work we apply a statistical analysis to the results of the first part in order to spot structural differences in the topological data. Interestingly, we find that the persistent entropy is a good statistical indicator of the topological differences in the functional brain networks obtained from a population of participants with ISAD and healthy controls. Although the relatively small sample size inevitably limits the generalizability of our findings, we argue that, to our knowledge, this is the first clear indication of differences in the structure of functional brain networks from ISAD and control adolescents.

## Materials and methods

### Datasets

The datasets used consist of resting-state correlation matrices from a population of 43 teenagers (24 with Inhaled Substance Abuse Disorder, 18 males, and 19 controls, 10 males), age range: 13-17 years. ISAD participants were recruited by contacting a Youth Integration Center in Mexico City from January 20, 2014 to June 30, 2018. Controls were recruited from high and senior high schools, which were all in Mexico City. All the procedures of the protocol were approved by both the Scientific Research Committee of the Centers for Youth Integration and the Ethics Committee of the Children’s Hospital of Mexico “Federico Gomez”. Parents/guardians have given written informed consent and adolescents assent to participate. The correlation matrices are available at https://doi.org/10.6084/m9.figshare.21941681.

### Data acquisition

The resting-state functional magnetic resonance images (eyes open, fixated on a cross) were acquired in a Siemens Magnetom Skyra 3T scanner 64-channel head coil. Multiband rs-fMRI (TR/TE/Flip Angle = 720ms /29s /44°, 48 slices, multiband accelerate factor = 8, matrix = 82 × 82, FOV = 268mm, voxel size = 3 × 3 × 3mm^3^, scan duration 6min i.e. 500 vol) was performed. Additionally, a high resolution T1-weighted anatomical image 3DMPRAGE (TR/TE = 2200ms /2.45ms, voxel size = 1mm^3^, iPAT = 2) was acquired.

### Preprocessing

Results included in this manuscript come from preprocessing performed using *fMRIPrep* 1.5.2 ([18, 19]; RRID:SCR_016216), which is based on *Nipype* 1.3.1 ([20]; [21]; RRID:SCR_002502).

#### Anatomical data preprocessing

The T1-weighted (T1w) image was corrected for intensity non-uniformity (INU) with N4BiasFieldCorrection [22], distributed with ANTs 2.2.0 [23, RRID:SCR_004757], and used as T1w-reference throughout the workflow. The T1w-reference was then skull-stripped with a *Nipype* implementation of the antsBrainExtraction.sh workflow (from ANTs), using OASIS30ANTs as target template. Brain tissue segmentation of cerebrospinal fluid (CSF), white-matter (WM) and gray-matter (GM) was performed on the brain-extracted T1w using fast [24, FSL 5.0.9, RRID: SCR_002823,]. Volume-based spatial normalization to one standard space (MNI152NLin2009cAsym) was performed through nonlinear registration with antsRegistration (ANTs 2.2.0), using brain-extracted versions of both T1w reference and the T1w template. The following template was selected for spatial normalization: *ICBM 152 Nonlinear Asymmetrical template version 2009c* [[25], RRID:SCR_008796; TemplateFlow ID: MNI152NLin2009cAsym].

#### Functional data preprocessing

For each of the 1 BOLD runs found per subject (across all tasks and sessions), the following preprocessing was performed. First, a reference volume and its skull-stripped version were generated using a custom methodology of *fMRIPrep*. The BOLD reference was then co-registered to the T1w reference using flirt [26, FSL 5.0.9,] with the boundary-based registration [27] cost-function. Co-registration was configured with nine degrees of freedom to account for distortions remaining in the BOLD reference. Head-motion parameters with respect to the BOLD reference (transformation matrices, and six corresponding rotation and translation parameters) are estimated before any spatiotemporal filtering using mcflirt [28, FSL 5.0.9,]. The BOLD time-series (including slice-timing correction when applied) were resampled onto their original, native space by applying the transforms to correct for head-motion. These resampled BOLD time-series will be referred to as *preprocessed BOLD in original space*, or just *preprocessed BOLD*. The BOLD time-series were resampled into standard space, generating a *preprocessed BOLD run in [‘MNI152NLin2009cAsym’] space*. First, a reference volume and its skull-stripped version were generated using a custom methodology of *fMRIPrep*. Several confounding time-series were calculated based on the *preprocessed BOLD*: framewise displacement (FD), DVARS and three region-wise global signals. FD and DVARS are calculated for each functional run, both using their implementations in *Nipype* [29, following the definitions by]. The three global signals are extracted within the CSF, the WM, and the whole-brain masks. Additionally, a set of physiological regressors were extracted to allow for component-based noise correction [30, *CompCor*,]. Principal components are estimated after high-pass filtering the *preprocessed BOLD* time-series (using a discrete cosine filter with 128s cut-off) for the two *CompCor* variants: temporal (tCompCor) and anatomical (aCompCor). tCompCor components are then calculated from the top 5% variable voxels within a mask covering the subcortical regions. This subcortical mask is obtained by heavily eroding the brain mask, which ensures it does not include cortical GM regions. For aCompCor, components are calculated within the intersection of the aforementioned mask and the union of CSF and WM masks calculated in T1w space, after their projection to the native space of each functional run (using the inverse BOLD-to-T1w transformation). Components are also calculated separately within the WM and CSF masks. For each CompCor decomposition, the *k* components with the largest singular values are retained, such that the retained components’ time series are sufficient to explain 50 percent of variance across the nuisance mask (CSF, WM, combined, or temporal). The remaining components are dropped from consideration. The head-motion estimates calculated in the correction step were also placed within the corresponding confounds file. The confound time series derived from head motion estimates and global signals were expanded with the inclusion of temporal derivatives and quadratic terms for each [31]. Frames that exceeded a threshold of 0.5 mm FD or 1.5 standardised DVARS were annotated as motion outliers. All resamplings can be performed with *a single interpolation step* by composing all the pertinent transformations (i.e. head-motion transform matrices, susceptibility distortion correction when available, and co-registrations to anatomical and output spaces). Gridded (volumetric) resamplings were performed using antsApplyTransforms (ANTs), configured with Lanczos interpolation to minimize the smoothing effects of other kernels [32]. Non-gridded (surface) resamplings were performed using mri_vol2surf (FreeSurfer). The remaining components were dropped from consideration. Despiking was performed with AFNI’s 3DDESPIKE utility and the 36 parameters from the global confound regression.

Many internal operations of *fMRIPrep* use *Nilearn* 0.5.2 [33, RRID:SCR_001362], mostly within the functional processing workflow. For more details of the pipeline, see the section corresponding to workflows in *fMRIPrep*’s documentation.

### Code accessibility

We used Matlab for the statistical computations and Python for the construction of the filtration. All the calculations were performed on an AMD FX-8320E CPU 3.2 @ × 8 with Ubuntu 20.04 LTS 64 bits and 8GB of RAM. As finding the maximal clique is a computationally-expensive task, the filtration was only constructed up to dimension 2, which is sufficient for computations in 0 and 1-dimensional homology. For persistent homology the software Dionysus 2 was used in its Python version. All the scripts used are available in the Github project https://github.com/MartinMij/TDA-of-networks.

### TDA

Given a weighted graph *G*, a sequence of nested simplicial complexes, called a *filtration* of simplicial complexes, can be obtained in order to extract topological features by means of persistent homology. As a first step, usually one constructs the so-called *graph filtration of G*, first introduced in [6] (see also [9, 34]). This is a sequence of subgraphs of *G* where the first subgraph consists only of the vertices with no edges and gradually we add the more lightweight edges until reaching the complete *G*. An alternative way to proceed, first proposed in [34], is to consider the opposite direction, that is, first adding the edges with a greater weight. In this work we take this second approach, which can be described more formally as follows. Given a weighted graph *G*, let *δ*_max_ and *δ*_min_ correspond to the maximum and the minimum weights of the edges of *G*, respectively, and let us consider the ordered sequence of weights

$$\begin{array}{c} \delta_{\text{max}}=\delta_{1}>\delta_{2}>\cdots >\delta_{n-1}>\delta_{n}=\delta_{\text{min}}. \end{array}$$

Using these weights, we construct a sequence of nested subgraphs

$$\begin{array}{c} G_{\delta_{0}}\subset G_{\delta_{1}}\subset \cdots \subset G_{\delta_{n-1}}\subset G_{\delta_{n}}=G\;, \end{array}$$ (1)

where $G_{\delta_{0}}$ consists of the vertices of *G* with no edges and each $G_{\delta_{i}}$ with *i* ≥ 1 consists of the vertices of *G* and all the edges with weight greater than or equal to *δ*_*i*_. With the aim of obtaining comparable results from networks with different threshold scales, an important point, following [8], is to re-index the graph filtration: let *ρ*_*i*_ ∈ [0,1] be the density of the graph $G_{\delta_{i}}$, that is, the number of edges in $G_{\rho_{i}}$ divided by the number of edges in the complete graph *G*. In order to avoid confusion with the subscripts of the two filtrations, we denote by $G_{\rho_{i}}^{\prime }$ the graph $G_{\delta_{i}}$ where the index *ρ*_*i*_ is the density of $G_{\delta_{i}}$. With this notation, we can rewrite the graph filtration (1) as

$$\begin{array}{c} G_{\rho_{0}}^{\prime }\subset G_{\rho_{1}}^{\prime }\subset \cdots \subset G_{\rho_{n-1}}^{\prime }\subset G_{\rho_{n}}^{\prime }=G\;. \end{array}$$ (2)

The sequence begins with $G_{\rho_{0}}^{\prime }=G_{0}^{\prime }$, which contains all the vertices of *G* but has no edges, it continues with subgraphs obtained by gradually adding the heaviest edges, and it ends with $G_{\rho_{n}}^{\prime }=G_{1}^{\prime }$, which contains all the edges of *G*.

On the other hand, given any graph *G*, we have an associated simplicial complex *C*(*G*) with the information of the complete subgraphs, which is called the *clique complex*. The vertices of *C*(*G*) are the same as the vertices of *G* and the simplices of *C*(*G*) are the complete subgraphs of *G*. Thus, the graph filtration (2) has an associated filtration of simplicial complexes, which we call the *clique filtration*

$$\begin{array}{c} C_{\rho_{0}}\subset C_{\rho_{1}}\subset \cdots \subset C_{\rho_{n-1}}\subset C_{\rho_{n}}\;, \end{array}$$ (3)

where we denote by $C_{\rho_{i}}$ the clique complex $C(G_{\rho_{i}}^{\prime })$ for brevity.

Finally, by applying persistent homology to the clique filtration, we obtain barcodes, namely, multisets of intervals that represent the points along the filtration where certain topological feature lives. More precisely, given a filtration *F* of simplicial complexes, for any p∈N, its corresponding *p-persistent barcode* is the multiset

$$\begin{array}{c} Bc_{p}\left(F\right)=\left\{\left[x_{i},y_{i}\right)|1\leq i\leq r,\;\;r\in N\;\right\}, \end{array}$$

where each interval corresponds to a bar starting at *x*_*i*_ and ending at *y*_*i*_. For instance, each bar in a 0-dimensional barcode represents a connected component and its length is the ‘time’, as measured by the parameter *ρ*, that it takes before it is linked to another component. Therefore, short bars are associated to components which merge (‘die’) quickly with other components along the filtration. On the other hand, bars in a 1-dimensional barcode represent cycles (1-dimensional holes) and their length means their lifespan along the filtration, that is, the ‘time’ that it takes before they get filled.

**Fig 1:**
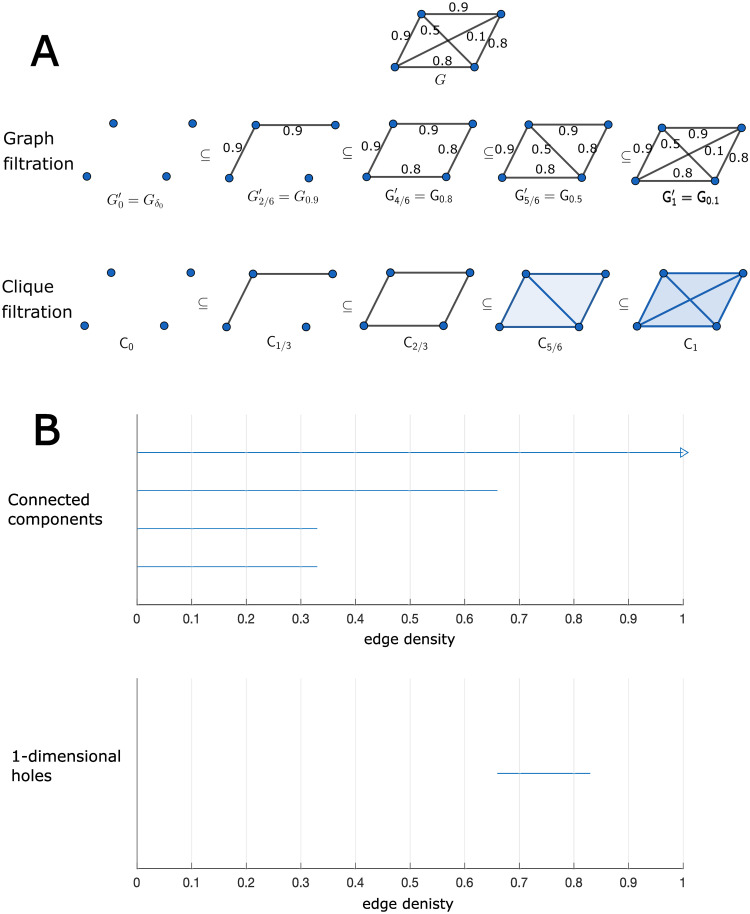
Graph filtration and their barcodes. Given a weighted graph *G*, we construct the corresponding clique filtration and the related barcodes. A: Given *G*, we construct first the graph filtration. For each graph in the filtration we build the associated clique complex, thus obtaining the clique filtration. Note that in the *G*-notation the index is the threshold, while in the *G*^′^-notation it is the edge density. B: 0- and 1-persistent barcodes obtained from the clique filtration. The first one represents the connected components along the filtration, while the second one the 1-dimensional holes.

In Fig 1 we provide a simple example of a graph, together with its graph filtration and the related 0- and 1-persistent barcodes.

### Persistent entropy

Persistent entropy is a commonly-used technique to map barcodes to real numbers. Given a *p*-persistent barcode *Bc*_*p*_(*F*), we first set *l*_*i*_ = *y*_*i*_ − *x*_*i*_ if *y*_*i*_ is finite or *l*_*i*_ = *m* + 1 − *x*_*i*_ if *y*_*i*_ is infinite, with *m* = max{*y*_*i*_|*y*_*i*_*isfinite*}. Intuitively, *l*_*i*_ is the duration (‘life’) of the *i*-th bar along the filtration. Then we define the distribution of bar lengths $p_{i}=\frac{l_{i}}{L}$, for *i* = 1, …, *r*, with *r* the total number of bars. Finally, the *p-persistent entropy* is defined as the (Shannon) entropy of the distribution of bar lengths, namely

$$\begin{array}{ccc} e_{p}\left(F\right) & ≔ & -\sum_{i=1}^{r}p_{i}\;\text{log}\left(p_{i}\right). \end{array}$$ (4)

Intuitively, the persistent entropy accounts for the variability of the lengths of the bars. Higher values of *e*_*p*_(*F*) signify that the lengths of the bars are more uniform. As proved in [35], the persistent entropy is stable in the sense that a small change in the barcode (with respect to the bottleneck distance) induces a small change in the persistent entropy. This is a very important property when dealing with data, as it implies that in case there is noise in the data, this will not affect the final results obtained from the persistent entropy analysis.

## Results

We analyzed 43 correlation matrices obtained from rs-fMRI data (see Materials and methods). The first 19 matrices correspond to healthy controls and the remaining 24 to a sample of inhaled substance abuse disorder (ISAD) participants. We consider these matrices as the adjacency matrices of weighted networks. So we can apply to these networks the ideas given in the ‘TDA’ and ‘Persistent entropy’ sections.

### TDA of control and ISAD subjects

**Fig 2:**
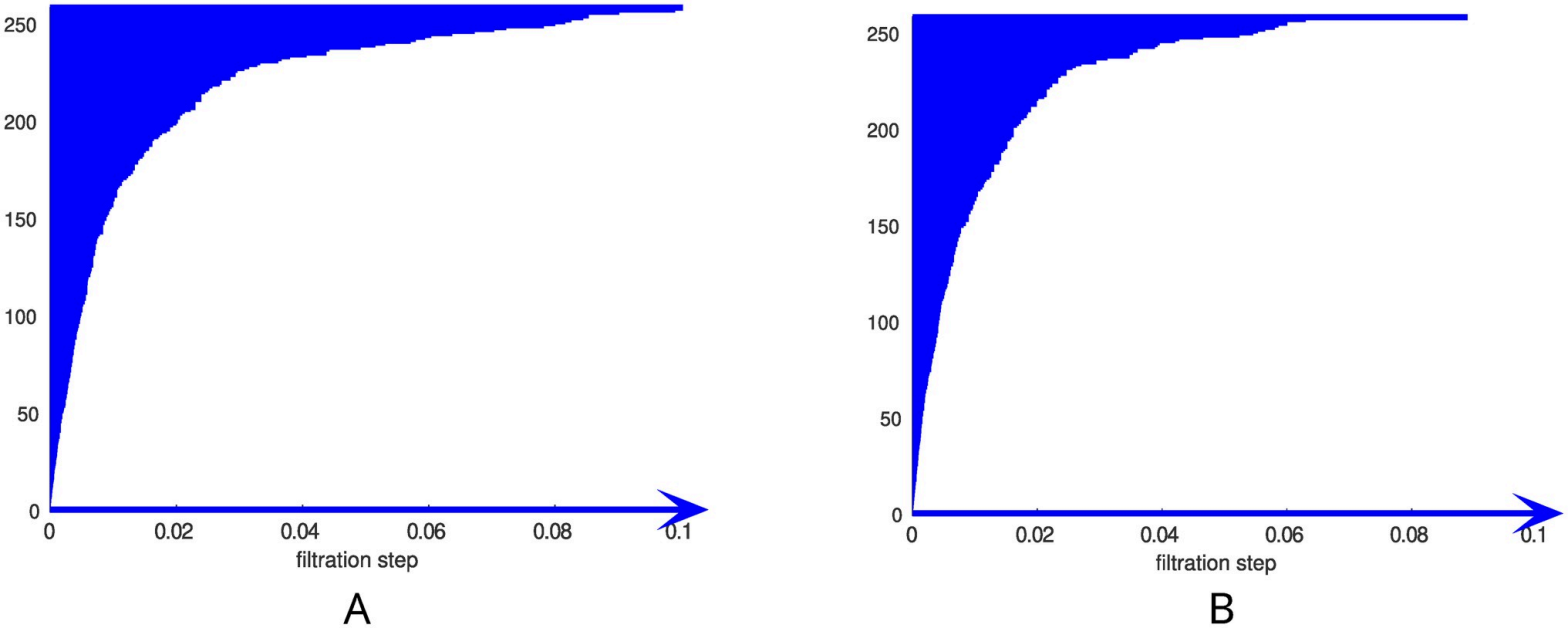
0-dimensional barcodes. A: 0-dimensional barcode associated to a control subject. B: 0-dimensional barcode associated to an ISAD subject.

Fig 2 shows the 0-dimensional barcodes from a representative control subject and an ISAD subject, respectively. The horizontal axis represents the filtration parameter, which in our case is the density *ρ* of the corresponding subgraph *G*_*ρ*_, while the vertical axis shows a number which labels the bars (connected components) in *G*_*ρ*_. The filtration begins at *ρ* = 0, which means that there are no edges, only 259 disconnected vertices. As *ρ* increases, corresponding to adding edges to the subgraph, some connected components merge. When two connected components merge, one of them disappears from the plot and its bar ends (‘it dies’). The longer a bar lives, the longer it takes for the corresponding connected component to merge with another. At the end of the filtration, for *ρ* = 1, we have only one connected component (because the graph *G* is connected). Therefore we expect that for large values of *ρ* only one component survives. Actually we observe in panel A of Fig 2 that, for a control subject, at *ρ* = 0.1 all the connected components but one have already died. This means that by considering approximately 10% of the heaviest edges, the corresponding subgraph is already formed by a single connected component, while at densities of about *ρ* = 0.08 there are still a few unmerged components. On the contrary, in panel B of Fig 2 we see that for a representative ISAD subject the last component is merged at approximately *ρ* = 0.08, and that most of the components are already merged at about 6% of the density.

**Fig 3:**
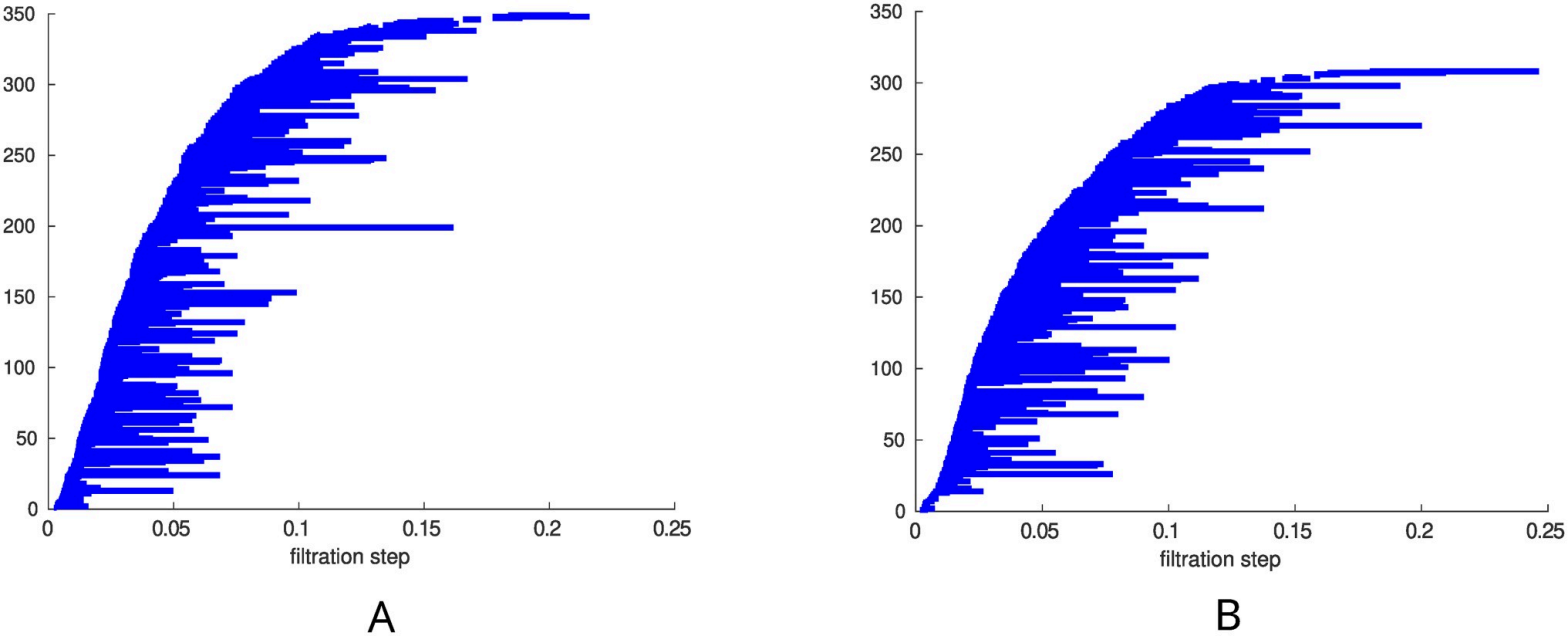
1-dimensional barcodes. A: 1-dimensional barcode associated to a control subject. B: 1-dimensional barcode associated to an ISAD subject.

Fig 3 shows the 1-dimensional barcodes from a representative control and ISAD subject. The horizontal axis is still the filtration parameter (density) *ρ*, which in these plots ranges from 0 to 25%, and the vertical axis still shows a number which labels the bars in *G*_*ρ*_. However, in this case the bars represent 1-dimensional holes in the graph, that is, *n*-vertex cycles, with *n* ≥ 4. Clearly there are no 1-dimensional holes at *ρ* = 0 (only isolated vertices). This is why the bars in the plot begin to appear (‘are born’) at higher densities, corresponding to when *n*-vertex cycles form. On the contrary, whenever an edge is added inside a 4-vertex cycle, this becomes filled with triangles and the bar ends (‘it dies’). Panel A in Fig 3 shows that for a control subject at densities greater than 20% there are no 1-dimensional holes. On the other hand, for an ISAD subject we can have 1-dimensional holes up to densities of 25%, as shown in panel B of Fig 3.

### Persistent entropy of control and ISAD subjects

**Fig 4:**
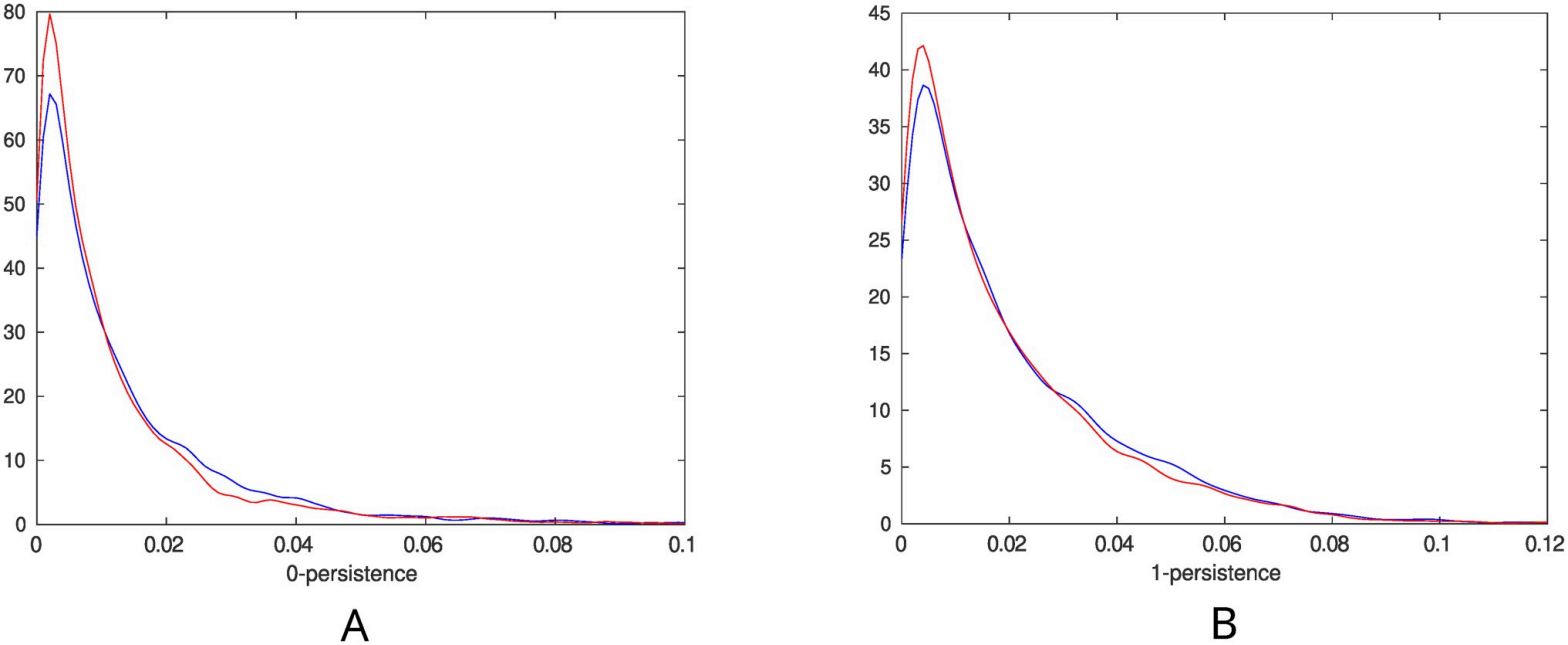
Distribution of the bar lengths. In blue the controls and in red the ISAD subjects. A: Distribution of the bar lengths in dimension 0. B: Distribution of the bar lengths in dimension 1.

To each subject we associate a network and then obtain a barcode through persistent homology, following the pipeline indicated in the Materials and Methods section. We thus generate four sets where each one consists of the collection of the lengths of all the bars of all the subjects corresponding to each dimension (0 and 1) and each population (ISAD and controls). A Lilliefors test showed that there is no evidence that these data sets are sampled from normal distributions, and therefore we have used a Kolmogorov-Smirnov (KS) test to show that the distributions of the bar lengths are different. For this, we used the two-sample KS test, as implemented in the kstest2 function in MATLAB. Fig 4 shows the distributions of bar lengths for the 0- and 1-dimensional persistence diagrams of each population. The KS test confirmed that in both cases (0- and 1-dimensional) the samples from the two groups belong to different distributions (for the 0-persistence, panel A of Fig 4, we obtained the KS statistic *D* = 0.0531 and *p*-value = 3.764 × 10^−7^, while for 1-persistence, panel B of Fig 4, we obtained the KS statistic *D* = 0.0327 and *p*-value = 0.0018).

In order to quantify the differences between the corresponding diagrams, we have further computed different statistical descriptors of these distributions of lengths of the bars, such as persistent entropy. Interestingly, we found that the persistent entropy is statistically different between the two groups, as detailed in the following.

**Fig 5:**
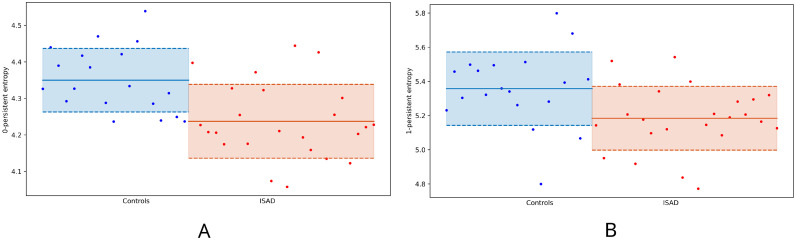
0- and 1-dimensional persistent entropy. Blue dots represent the value of the persistent entropy for the control subjects and red dots of the ISAD subjects. We see in panel A that the mean of the 0-persistent entropy of the ISAD population is lower than that of the controls. We see in panel B that the same happens when considering the mean of the 1-persistent entropy.

Fig 5 shows the 0- and 1-persistent entropy values of each subject. The blue dots correspond to the control group and the red ones to the ISAD group. We also show the mean of the persistent entropy within each group, with the corresponding standard error intervals. For the 0-persistent entropy we obtained: 4.346 ± 0.020 (controls) and 4.233 ± 0.021 (ISAD) (Panel A in Fig 5); and for 1-persistent entropy of 5.357 ± 0.050 (controls) and 5.184 ± 0.039 (ISAD) (Panel B in Fig 5). We conclude that, both in dimension 0 and 1, ISAD subjects have a lower entropy, with a large effect size (calculated with Cohen’s *d*) in both dimensions, 1.1681 and 0.8365, respectively. Here *d* is computed with the formula

$$\begin{array}{c} d=\frac{|{\bar{x}}_{1}-{\bar{x}}_{2}|}{\sqrt{\frac{s_{1}^{2}+s_{2}^{2}}{2}}} \end{array}$$

where ${\bar{x}}_{1}$ and *s*_1_ are respectively the mean and standard deviation of the persistent entropy of the control group and ${\bar{x}}_{2}$ and *s*_2_ the mean and standard deviation of the persistent entropy of the ISAD group. A Lilliefors test (lillietest function implemented in MATLAB) indicated that the 0-persistent entropy of controls (*D* = 0.1469, *p*-value = 0.3288), 1-persistent entropy of controls (*D* = 0.1382, *p*-value = 0.4169), 0-persistent entropy of ISAD subjects (*D* = 0.1476, *p*-value = 0.1647) and 1-persistent entropy of ISAD subjects (*D* = 0.1220, *p*-value = 0.0.4127) are normally distributed. Therefore, we proceeded further to corroborate that the two distributions of persistent entropy for each group are different (in each dimension) by applying a two-sample *t*-test. For the 0-persistent entropy we obtained a *t*-statistic of 3.835 and a *p*-value of 4.284 × 10^−4^, while for the 1-persistent entropy we obtained a *t*-statistic of 2.701 and a *p*-value of 0.0104.

Furthermore, in order to account for potential confounding variables such as age and sex, we have run a multiple linear regression analysis (using the OLS Python function of the statsmodels module) with the 0-persistent entropy *e*_0_ and 1-persistent entropy *e*_1_ as independent variables and age, sex and diagnostic (control or ISAD) as dependent variables with the model

$$\begin{array}{c} e_{i}=\beta_{0}+\beta_{1}X_{1}+\beta_{2}X_{2}+\beta_{3}X_{3}, \end{array}$$

where the variable sex is represented as *X*_1_, age as *X*_2_ and diagnostic as *X*_3_. In Tables 1 and 2 we display the results of the linear regression analysis. In both cases the correlation between the variables age and sex with the persistent entropy is not statistically significant (*p*-value>0.05), while, as expected, there is evidence of a non-zero correlation between the persistent entropy and the diagnostic variable in both dimensions (*p*-values of 0.0016 and 0.0184 for the 0- and 1-persistent entropy, respectively). These findings suggest that the persistent entropy is mainly affected by whether there is an abuse of inhaled substances or not.

**Table 1:** Linear regression results for the 0-persistent entropy.

Variable	Coefficient (*β*)	Std. error	t	p-value
Intercept	0.5747	0.0727	7.9056	<0.0001
Sex (male = 0, female = 1)	0.0712	0.0673	1.0587	0.2963
Age (years)	-0.0029	0.0891	-0.0330	0.9738
Diagnostic (control = 0, ISAD = 1)	-0.2178	0.0644	-3.3817	0.0016

Regression corresponding to *e*_0_ as independent variable, *n* = 43, *R*^2^ = 0.278, adjusted *R*^2^ = 0.223.

**Table 2:** Linear regression results for the 1-persistent entropy.

Variable	Coefficient (*β*)	Std. error	t	p-value
Intercept	0.5458	0.0724	7.5427	<0.0001
Sex (male = 0, female = 1)	0.0461	0.0670	0.6890	0.4948
Age (years)	0.0077	0.0887	0.0869	0.9311
Diagnostic (control = 0, ISAD = 1)	-0.1577	0.0641	-2.4598	0.0184

Regression corresponding to *e*_1_ as independent variable, *n* = 43, *R*^2^ = 0.166, adjusted *R*^2^ = 0.102.

**Fig 6:**
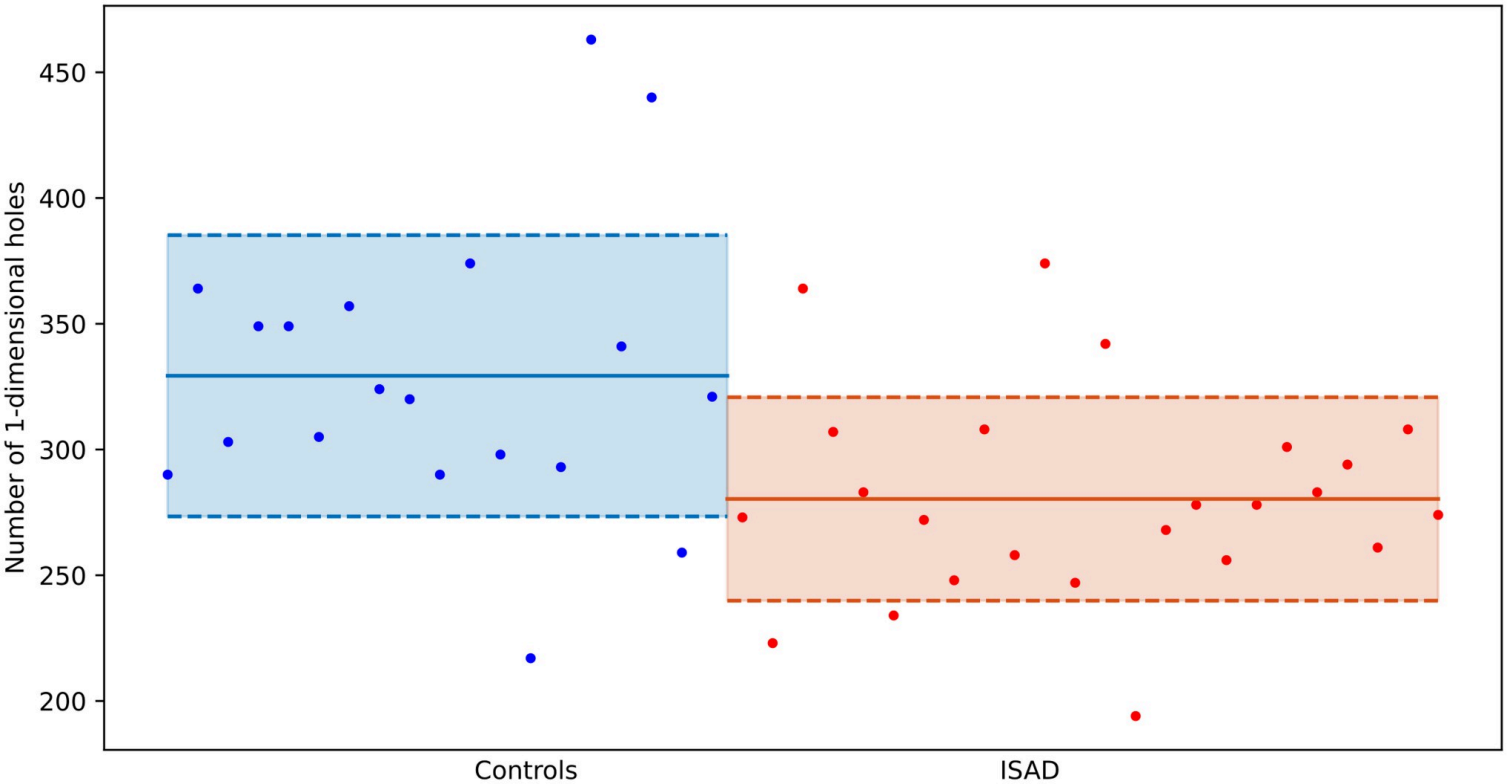
1-dimensional holes. Total number of bars in the 1-dimensional barcodes of each subject.

The difference in the persistent entropy can be ascribed in general to two factors: on the one hand it could be that a distribution has a larger entropy because it has access to a larger number of states; on the other hand, if the number of possible states is the same, then the distribution with a larger entropy is the most uniform one. The 0-dimensional barcodes have always the same total number of bars (259), and therefore in this case the fact that the persistent entropy of the controls is larger than that of the ISAD subjects has to be ascribed to the fact that they have a more uniform distribution of the lengths of the bars. In the 1-dimensional case, this difference is due to the fact that 1-dimensional barcodes of the control group have more possible states than those of the ISAD group, or in other words, more 1-dimensional holes appear in the control networks (see Fig 6). Interestingly, as shown in panel B of Fig 4, these 1-dimensional holes are more likely to be long-lived in the control case compared to the ISAD case.

## Discussion and conclusions

In this work we have shown that the persistent entropy, a tool from topological data analysis, can be helpful in spotting topological differences in the functional brain networks obtained from a population composed by participants with inhaled substance abuse disorder (ISAD) and healthy controls. Indeed, the average 0- and 1-persistent entropy of the control subjects is always larger than that of the ISAD subjects and, moreover, a statistical analysis reveals that the two distributions corresponding to the two groups are significantly different in both dimensions.

The case of the 0-persistent entropy is more interesting because it directly implies that the bar length distribution of the controls is more uniform than that of the ISAD subjects, as can also be seen in panel A of Fig 4, where we notice that the peak of the ISAD distribution is sharper and it happens at shorter lengths. This means that, although most of the connected components of both populations are short-lived, ISAD’s bars tend to be shorter than the ones of the controls. This suggests that the controls are inclined to develop a more robust network in the following sense. Considering the growing process outlined in the TDA paragraph of the Materials and Methods section (where the initial graph consists only of vertices and the heaviest edges are added consecutively), longer bars imply that newly added edges are more likely to connect vertices within a connected component rather than vertices in two different connected components. In this sense, at low density, removing an arbitrary edge in an ISAD network is more likely to disconnect the network than in a control one.

For the above reasons we argue that the lower value of the 0 − persistent entropy of the ISAD population shall be associated with the construction of less paths between any two given points, which implies a more vulnerable (functional) network.

The fact that the control subjects typically have higher values of the 1 − persistent entropy could be further associated with their ability to access a larger repertoire of functional states [36]. This will be an interesting direction for further research.

Finally, our results are consistent with the previous analysis performed in [37]. There, a graph-theoretical analysis based on the network efficiency, together with a clinical assessment with standard tests such as the Working Memory Index from the Wechsler intelligence scale, the Wisconsin Card Sorting Test, the Stroop test and the Tower of Hanoi test, have shown that the lower degree of working memory, mental flexibility, inhibitory control and sequential planning found in the ISAD group can be associated with the lower level of connectivity efficiency in the default mode network, the salience network, and the fronto-parietal network.

Although the sample size is small, the effect size is large in both 0- and 1-entropies. Nevertheless, the conclusions must be taken with care and further studied. We argue that, from the combined analysis of our results and those of [37], which were obtained with common graph measures, it stands out that the reduced robustness in ISAD’s functional network might be responsible for the alterations in the cognitive function of ISAD subjects. Further research in this direction, analyzing the role of different sub-networks and their individual impact on the persistent entropy analysis, would bring a deeper understanding on the role of the connectivity of the network and will be performed in future work.
